# Inhibitory and Apoptosis-Inducing Effects of Newcastle Disease Virus Strain AF2240 on Mammary Carcinoma Cell Line

**DOI:** 10.1155/2015/127828

**Published:** 2015-03-02

**Authors:** Umar Ahmad, Ismaila Ahmed, Yong Yoke Keong, Nizar Abd Manan, Fauziah Othman

**Affiliations:** ^1^Research Laboratory of Anatomy & Histology, Faculty of Medicine and Health Sciences, Universiti Putra Malaysia (UPM), 43400 Serdang, Selangor, Malaysia; ^2^Department of Human Anatomy, Faculty of Medicine, Bauchi State University, PMB 65, Gadau, Nigeria; ^3^Department of Human Anatomy, Faculty of Medicine and Health Sciences, Universiti Putra Malaysia (UPM), 43400 Serdang, Selangor, Malaysia

## Abstract

Breast cancer is the malignant tumour that developed from cells of the breast and is the first leading cause of cancer death among women worldwide. Surgery, radiotherapy, and chemotherapy are the available treatments for breast cancer, but these were reported to have side effects. Newcastle disease virus (NDV) known as *Avian paramyxovirus* type-1 (APMV1) belongs to the genus *Avulavirus* in a family Paramyxoviridae. NDV is shown to be a promising anticancer agent, killing tumour cells while sparing normal cells unharmed. In this study, the oncolytic and cytotoxic activities of NDV AF2240 strain were evaluated on MDA-MB-231, human mammary carcinoma cell line, using MTT assay, and its inhibitory effects were further studied using proliferation and migration assays. Morphological and apoptotic-inducing effects of NDV on MD-MB-231 cells were observed using phase contrast and fluorescence microscopes. Detection of DNA fragmentation was done following terminal deoxyribonucleotide transferase-mediated Br-dUTP nick end labeling staining (TUNEL) assay, which confirmed that the mode of death was through apoptosis and was quantified by flow cytometry. Furthermore, analysis of cellular DNA content demonstrated that the virus caused an increase in the sub-G1 phase (apoptotic peak) of the cell cycle. It appears that NDV AF2240 strain is a potent anticancer agent that induced apoptosis in time-dependent manner.

## 1. Introduction

Breast cancer comprised 23% of all cancers in women and is the commonest malignancy that causes cancer mortality in women [1]. Studies have identified few biological and lifestyle, behavioral as risk factors associated with an increased breast cancer development. These include inherited genetic mutations of* BRCA1* and* BRCA2* genes, and family with personal history of breast cancer, hormonal, diet, and environmental factors [2, 3]. The conventional approach to the treatment of cancer is cytotoxic chemotherapy, either alone or in combination with surgery and radiotherapy. Goldhirsch et al. [4] reported that the conventional methods of treatment are usually painful and are often accompanied with many complications such as endometriosis, blood clots, vomiting, and hair loss. Recently, viral therapy for cancer (virotherapies) is known to have potential in cancer treatment, as some viruses have been found with oncolytic properties, having the ability to suppress cancer tumour. Virotherapy involves the treatment of cancer by using viruses specifically to infect cancer cells while leaving normal cells unharmed [5]. These viruses infect, replicate in, and kill human cancer cells through diverse mechanisms [6]. Newcastle disease virus (NDV) is one of such oncolytic viruses that replicate and kill cancer cells while sparing normal cells. NDV is a member of the new genus* Avulavirus* within the family Paramyxoviridae. The virus causes a highly contagious disease affecting brain and gastrointestinal and respiratory tracts of a poultry species [7]. However, it results in mild conjunctivitis, laryngitis, and influenza-like systems when exposed to humans [8]. Interest in the use of the oncolytic NDV to kill cancer was due to its specification in targeting cancer cells without causing excessive damage to healthy normal cells. It was reported that this therapy is well tolerated, and no serious side effects have been observed in any of the trials [9–11]. Thus, NDV is used as antineoplastic and immunostimulatory agent in clinical tumor therapy.

Several strains of NDV such as 73-T, HUJ, PV701, and MTH68 have been shown to exhibit similar oncolytic properties as those of NDV AF2240 strain [12–14]. Further to this, additional exploration of the two Malaysian oncolytic NDV strains, AF2240, and V4, have also been studied on allografted 4T1 breast cell line* in vivo* [15] and on WEHI-3B leukaemic cell line and DBTRG.05MG human glioblastoma cells* in vitro* [16, 17]. Of all these strains, only AF2240 (velogenic) was found to be more effective and showed better cytotoxic effect on* in vitro* MCF-7 cells as compared to the V4-UPM (lentogenic) strain [18]. Thus, AF2240 strain has the most significant anticancer activity and had proven to be relatively effective in suppressing tumors growth through apoptosis induction [15, 19]. But the apoptosis-inducing effects for its oncolysis are not clearly understood. Apoptosis is an active programmed cell death, consisting of an essential sequence of physiological processes caused in response to specific stimuli [20]. Cell undergoing apoptosis showed some distinctive morphological and biochemical features. The morphological features can be recognized as cell shrinkage, membrane blebbing, and nuclear fragmentation into membrane-bound apoptotic bodies finally phagocytized by neighbouring cells [21], whereas the biochemical hallmark of apoptosis is characterized by DNA degradation or fragmentation of the internucleosomal DNA in which the genome is cleaved at internucleosomal sites, generating a “ladder” of DNA fragments when analyzed by agarose gel electrophoresis [22].

From the above literature search, the data were used to initiate further research, to investigate the effects of NDV AF2240 strain on human breast cancer cell lines in different strategic ways, targeting how it affect the DNA through fragmentation quantitatively. Furthermore, human endothelial cell lines were used to evaluate the antiangiogenic effects of the AF2240 using the two* in vitro* models of angiogenesis: proliferation and migration. Thus, the hypothesis of this study is that NDV AF2240 strain suppressed breast cancer growth by inhibiting proliferation, migration, and inducing apoptosis to cancer cells* in vitro*.

## 2. Materials and Methods

### 2.1. Propagation and Purification of NDV AF2240 Strain

The seed virus of the avirulent, nonlytic strain NDV AF2240 was obtained from Biologic Laboratory, Unit of Virology Laboratory, Faculty of Veterinary Medicine, Universiti Putra Malaysia (UPM), Serdang, Selangor. The virus strain was propagated in specific pathogens free (SPF) embryonated eggs of 9–11 days old at 37°C for 72 hours. The allantoic fluids were harvested and the presence of virus was confirmed by Haemagglutination test [23]. Purification of the virus was done as previously described by [24].

### 2.2. Cells and Cell Culture

The human mammary cancer cell line, MDA-MB-231, and the normal human fibroblast cells, Hs578Bst with Catalogue numbers* ATCC* Cat. HTB-26 and HTB-125 were purchased from the American Type Culture Collection (ATCC, Rockville, MD). While the EndoGRO human umbilical endothelial (HUVE) cell line Catalogue number SCCE001 (Merck Millipore, USA), was kindly donated by Dr. Yong Yoke Keong, Unit of Physiology, Department of Human Anatomy, Faculty of Medicine and Health Sciences, Universiti Putra Malaysia (UPM), Serdang, Selangor, Malaysia.

### 2.3. Cell Counting and MTT Cytotoxic Assay

Monolayer method of MTT assay was carried out using three different cell lines. MDA-MB-231, HUVECs, and Hs578Bst cells were grown in a 25 cm^2^ flask (Nunc, Denmark) until they reached 80–90% confluence. Cells were trypsinized and centrifuged and 1 mL specific media for each cell were added to resuspend the respective cells. About 10 *μ*L of the cell suspension was pipetted and mixed with 10 *μ*L trypan blue and then counted using haemocytometer (Neubauer, France). Each specific cell culture medium was added into the 50 mL tube to obtain the concentration of 1 × 10^5^ cells/mL. 100 *μ*L was pipetted into each well of the 96-well v-bottom plate (Nunc, Denmark). The cells were then incubated for 24 hours in a humidified incubator supplied with 5% CO_2_ at 37°C. After the incubation period, old media were pipetted out and discarded. Then, 50 *μ*L new media without supplements were added into each well. 50 *μ*L of the purified NDV AF2240 virus was added into the first column consisting of eight wells. The purified virus was resuspended then, 50 *μ*L from the first column was transferred into the second column of the wells and resuspended to make it be twofold dilution. This step was continued to get a series of dilutions from the highest to the lowest titre. For the control, PBS was added into the wells instead of the virus concentration and incubated for a period of 45 minutes. After that, each well was topped up with 150 *μ*L of supplemented culture media and the plate was further incubated for 72 hours.

After incubation period, 20 *μ*L (5 mg/mL PBS) of MTT solution was added into each well in the dark and the plate was covered with aluminium foil and then incubated for 4 hours in a humidified incubator. After 4 hours, all the media were removed and 100 *μ*L of 100% DMSO (AppliChem GmbH, Germany) was added into each well and left at room temperature for 30 minutes until the dark blue formazan crystals were dissolved. Absorbance was read at 570–590 nm using the enzyme-linked immunosorbent assay (ELISA) molecular device reader (EMax Endpoint, US). Percentage of viable cells was calculated using Mosman's [25] formula for percentage viability calculations:

(1) $$\begin{array}{c} \%\text{Viability}=\frac{\text{OD}\;\text{Sample}}{\text{OD}\;\text{Control}}\times 100, \end{array}$$

where OD means the optical density of the cells.

Graphs of percentage cell viability against the virus titre (HA Unit) were plotted for all the three cell lines: MDA-MB-231, HUVECs, and Hs578Bst. IC_50_ value was determined from the graphs that is, the concentration required to kill 50% of the treated cell's population as compared with the controls.

### 2.4. Quick Cell Proliferation Assay

The effects of Newcastle disease virus (NDV) AF2240 on the proliferation of MDA-MB231 cell line were assessed using Quick Cell Proliferation Colorimetric Assay Kit (BioVision, USA). Briefly, the cells were grown in a 25 cm^2^ until their confluent. Trypsinized cells were counted as previously described [26]. Cells were plated in 96-multiwell microtiter flat bottom plate (Nunc, Denmark) at a density of 1 × 10^4^ cells/mL in a final volume of 100 *μ*L/well in culture medium and then incubated for 24 hours to allow confluent. Old media were removed and replaced with a new media containing the graded concentration of the virus (twofold dilution). Some wells were left untreated but treated with PBS to serve as control. Triplicate experiments were run before the cells were incubated for 24 hours in a humidified 5% CO_2_ incubator at 37°C. After the end of this treatment period in incubation, 10 *μ*L of the lyophilized WST reagent was added to each well and further incubated for 3 hours in a standard culture conditions. The reaction mixture was stopped by adding 10 *μ*L of the stop solution into each well. The cultured cells were then shaked thoroughly for 1 minute. Finally, microtiter reader at 420–480 nm wavelengths measured the absorbance of the treated and untreated samples.

### 2.5. Cell Migration Assay

Cells were cultured in 6-well plates at a density of 2 × 10^5^ cells/mL in a serum-free DMEM medium for 24 hours. After the cells became confluent, the cell's monolayer was scratched horizontally with a tip of 200 *μ*L yellow pipette, to obtain a monolayer culture with space without cells as was described previously [27]. The scratched wound area in the plates was washed twice with PBS to remove the floating cells. Then, cells were incubated with medium containing 10% serum with or without IC_50_ concentration value of NDV AF2240 to serve as both positive and negative control cells, respectively. Cells migrating from the edge of the wound or scratched area were photographed at 0, 24, and 48 hours using phase contrast inverted microscope.

### 2.6. Phase Contrast Microscopic Study

The cellular morphology of human breast cancer cell line, MDA-MB231, was assessed and studied using phase contrast microscope after posttreatment period with Newcastle disease virus (NDV) AF2240. Cells were seeded into a 6-well plate (Nunc, Denmark) at concentration of 5 × 10^6^ cells/mL per well in a final volume of 2 mL/well culture medium. The cells were treated with IC_50_ concentration (25.99 HAU/mL) value of the virus and incubated in a humidified incubator at 37°C in an atmosphere of 5% CO_2_ for 24, 48, and 72 hours. The control cells (some wells) were left untreated to serve as a control. The changes in cellular morphology (features) were observed after the incubation period, using phase contrast microscope (Olympus, Japan).

### 2.7. Apo-BrdU* In Situ* DNA Fragmentation Assay

#### 2.7.1. Cells Seeding, Treatment, and Cells Fixation

The MD-MB-231 cells were seeded into a 6-well plate (Nunc, Denmark) at a cell density of 5 × 10^6^ cells/mL per well in a 2 mL culture medium for both the flow cytometry and fluorescence microscopic study, respectively. Cells were treated with IC_50_ concentration (25.99 HAU/mL) value of the virus and incubated for periods of 24, 48, and 72 hours in a humidified incubator at 37°C in an atmosphere of 5% CO_2_. Untreated cells were left with no virus treatment, serving as a control. Old media were discarded immediately, after the incubation periods and then the cells were washed with 1 mL of PBS. The cells were then trypsinized using EDTA 0.5% trypsin and incubated for few seconds to allow the cells to be detached from the well. Cell's pellet was obtained by adding a complete growth medium to each well to stop the activity of the trypsin and then centrifuge. Pelleted cells were resuspended in 0.5 mL of PBS and then with 5 mL of 1% (w/v) paraformaldehyde in PBS and placed on ice for 15 minutes. After that, cells were centrifuged for 5 minutes at 300 ×g and the supernatants were discarded. The obtained pellets were washed in 5 mL of PBS and pelleted by centrifugation. This washing and centrifugation steps were repeated twice and then resuspended in 0.5 mL PBS. At this stage, 5 mL of ice-cold 70% (v/v) ethanol was added to the cells and kept for 30 minutes on ice. Lastly, the cells were stored in 70% (v/v) ethanol at −20°C before use.

### 2.8. Flow Cytometry and Fluorescence Microscopic Study

The stored cells in 70% (v/v) ethanol were retrieved from −20°C freezer and thawed. The fixed cells were resuspended by swirling the vial and 1 mL of the cell suspension was aliquoted (≈1 × 10^6^ cells per mL) and put into 12 × 75 mm Falcon tubes. After that, the cells were centrifuged at 300 ×g for 5 minutes and the ethanol was removed carefully by aspiration with the pipette. 1 mL of wash buffer was added to resuspend the cells, followed by centrifugation as before, and the supernatants were aspirated carefully. This step was repeated twice. Then, the cells were resuspended in 50 *μ*L of the DNA Labeling Solution for each tube and incubated for 60 minutes at 37°C. In order to resuspend the cells, cells were shaked every 15 minutes during incubation period, after which, 1 mL of rinse buffer was added to each tube and centrifuged at 300 ×g for 5 minutes. The supernatants were removed by aspiration, and this rising and centrifugation steps were repeated once. Cells pellets were resuspended in 100 *μ*L Antibody Solution and incubated for 30 minutes in the dark at room temperature. To analyze the cells by flow cytometry, 0.5 mL propidium iodide/RNase A solution was added to each 12 × 75 mm Falcon tube and incubated in the dark for 30 minutes at room temperature. The cells were then analyzed at Ex/Em = 488/520 nm for FITC and 488/623 nm for PI using flow cytometer (Beckman Coulter, USA). However, for fluorescence microscopy study, 60 *μ*L PI/RNase A solution was added to each 12 × 75 mm Falcon tube, and incubated for 30 minutes in the dark at room temperature. After that, the cells were resuspended to make an individual cell suspension. Then, about 20 *μ*L of the mixture of cell suspension was sucked out and drop onto a slides before being covered with a cover slip. The slides were viewed under a fluorescence microscope (USA) using FITC and rhodamine filters (apoptotic cells showed green staining over and orange-red PI counter-staining). Viable and apoptotic cells were observed and quantified.

### 2.9. DNA Staining for Cell Cycle Analysis

Fixations of cells were done following the Apo-BrdU* in situ* DNA fragmentation assay kit (BioVision, USA) as mentioned previously. Briefly, cells were pelleted and fixed by adding 70% ice-cold ethanol and kept for 6 hours at −20°C. The fixed cells were resuspended and centrifuged at 300 ×g for 5 minutes and the supernatant was removed by aspiration and discarded. The pellets were washed with 1 mL wash buffer twice and the supernatant was aspirated and discarded. After that, the cells were stained with 50 *μ*L DNA Labeling Solution and incubated for a period of 60 minutes at room temperature. Cells were shaked for every 15 minutes within this period of incubation. Then, 0.1 mL of Antibody Solution was added to each 12 × 75 mm Falcon tube and incubated for 30 minutes in the dark at 37°C. Finally, 0.5 mL PI/RNase A solution was added and incubated for 30 minutes. The cell cycle was analyzed by flow cytometry (Beckman Coulter, USA).

### 2.10. Statistical Analysis

All data values represent at least three independent experiments and are expressed as mean ± SD. Significant levels between the groups were assessed and compared by one-way ANOVA following Turkey HSD Post Hoc multiple comparison tests. *P* values less than 0.05 were considered to be statistically significant.

## 3. Results

### 3.1. Result for Virus Propagation

Haemagglutination (HA) test was carried out immediately after propagation, clarification, and purification of the virus to determine the highest titre of the purified NDV AF2240 virus. The highest virus titre obtained was 2^8^ or 256 HA units.

### 3.2. Results for MTT Assays

**Figure 1:**
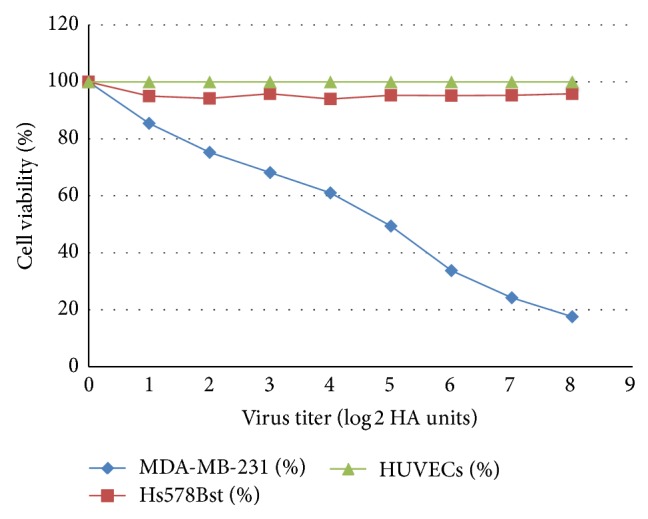
Scatter plot of the MTT Assay with regression line of NDV AF2240 IC_50_ (2^4.7^ HAU/mL) values determined in MDA-MB-231 cell lines (*y* = −10.251*x* + 98.206) after 72 hours of treatment. This result indicated the significant inhibitory effect of the virus on MD-MB-231, but no effect was observed on Hs578Bst and HUVEC cells. Data shown are mean ± SD of triplicate (*n* = 3) independent experiments. Correlation coefficient *R* ^2^ = 0.99176.

MTT Assays were carried out using the highest titre of the purified NDV AF2240 (stated above). The IC_50_ value of the virus was determined from this assay by plotting the graph of percentage cell viability against the virus tire, using MS Excel spreadsheet and the value obtained for MDA-MB-231 was 2^4.7^ or 25.99 HA units and there were no observable effects of this virus on human umbilical vein endothelial cells, HUVECs, and normal fibroblast of human breast cell lines, Hs578Bst (Figure 1). This shows that 2^4.7^ HA units of NDV AF2240 are needed to kill 50% of MDA-MB-231 cell population, whereas the virus does not have effects on HUVECs and Hs578Bst cell lines, respectively.

### 3.3. Quick Cell Proliferation Assay

#### 3.3.1. Antiproliferation of NDV AF2240 on MDA-MB-231 Cells

**Figure 2:**
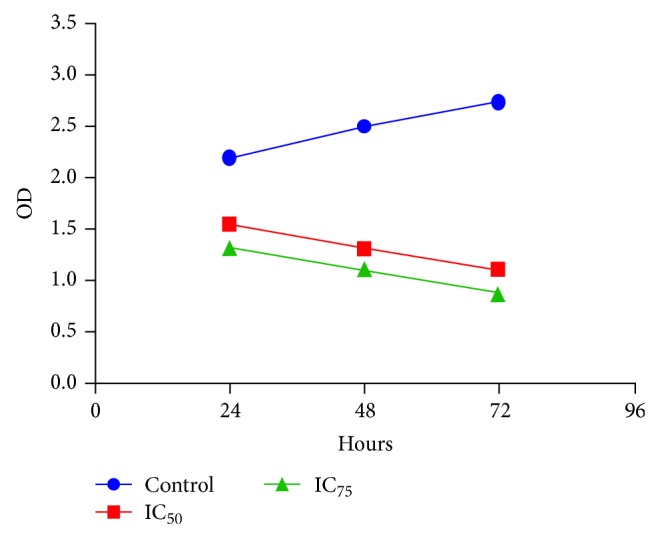
Quick Cell Proliferation Colorimetric Assay for IC_50_ and IC_75_ concentration values of the NDV AF2240 virus strain against MDA-MB-231 cells at 24, 48, and 72 hours after treatment. As indicated, the growth rates for the treated cells decreased significantly as compared with the control cells (*P* < 0.05). However, no significant different (*P* > 0.05) was observed between the two-virus concentrations.

To study the effect of NDV AF2240 virus strain on cell proliferation, MDA-MB-231 breast cancer cells were treated with virus titre at IC_50_ and IC_75_, respectively. Antiproliferative activity was evaluated after 24, 48, and 72 hours periods, following Quick Cell Proliferation Colorimetric Analysis Kit.  Figure 2 shows that NDV AF2240 inhibited the proliferation of MDA-MB-231 treated cells in both concentrations of the virus. As indicated, the growth rates decreased significantly in the treated cells as compared with the untreated cells (*P* < 0.05). High concentration (IC_75_) showed higher inhibition rate than the low concentration (IC_50_), but there was no significant different (*P* > 0.05) between the two concentrations.

**Figure 3:**
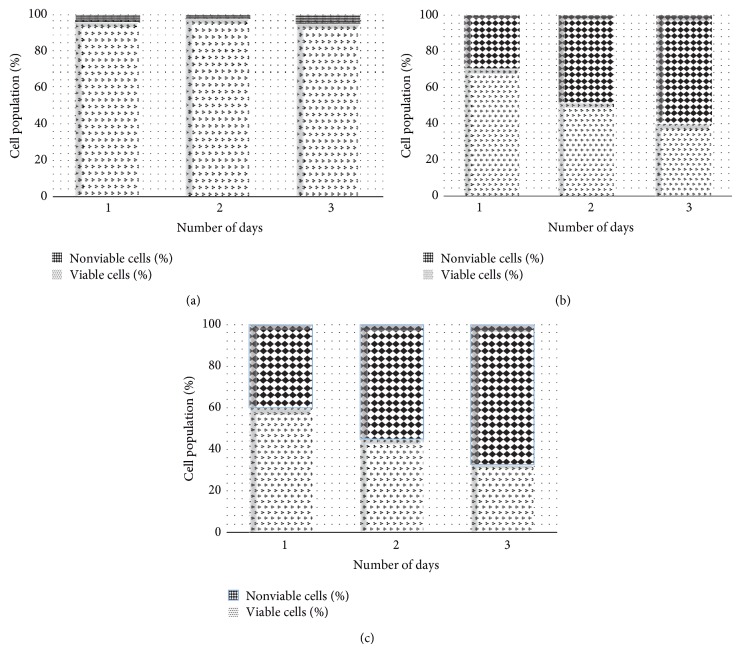
Percentage of viable and nonviable cells for MDA-MB-231 cell line exposed to time course. (a) Untreated (cancer) control cells, (b) cells treated with IC_50 _concentration value (25.99 HAU/mL) of NDV AF2240, and (c) cells treated with IC_75_ concentration (128 HAU/mL) of NDV AF2240. Results were expressed as mean (%) from triplicate experiments.

To determine if this inhibitory activity of the virus would lead to an increase in the percentage of cells death, a time-course study was conducted at day 1, day 2, and day 3.  Figure 3(a), being the control cells, demonstrated that the cells are intact with no significant number of death cells, whereas Figures 3(b) and 3(c) indicated that NDV AF2240 inhibited the proliferation of MDA-MB-231 breast cancer cell line by more than 59% and 67% at IC_50_ and IC_75_ after day 3, respectively. Approximately 29% and 40% inhibitions were obtained at IC_50_ and IC_75_ after day 1, respectively, whereas not less than 47% and 55% were obtained at IC_50_ and IC_75_ after day 2 of treatment on MDA-MB-231 cell lines, respectively. As shown in Figure 2, NDV virus suppressed this cancer growth in time-dependent manner. Therefore, it is suggested that NDV AF2240 virus strain exerts antiproliferative effects on MDA-MB-231 human breast cancer cell lines* in vitro*.

#### 3.3.2. Inhibition of MDA-MB-231 Cells Migration

**Figure 4:**
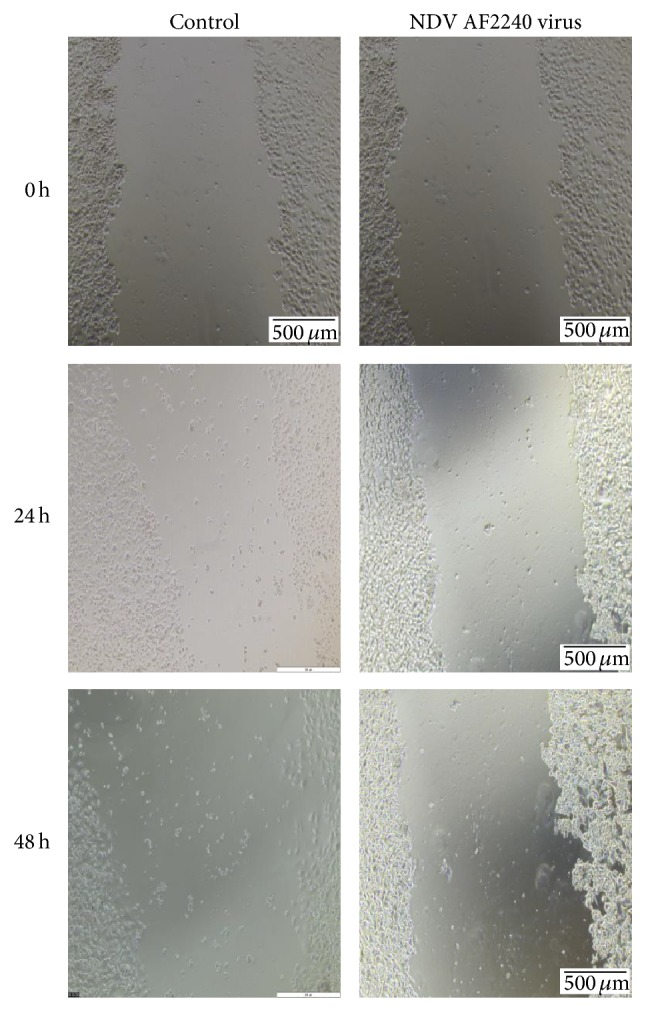
Effect of NDV AF2240 strain on cell migration by scratch wound assay. Confluent monolayers were cultured without (control) and with IC_50_ (25.99 HAU/mL) value of the virus and the migration was evaluated by wound healing assay at 24 and 48 h. Cells were photographs at 0, 24, and 48 hours after viral treatment. Note: at 48-hour time, the virus ruptured the cells and disjointed them from neighboring cells.

**Figure 5:**
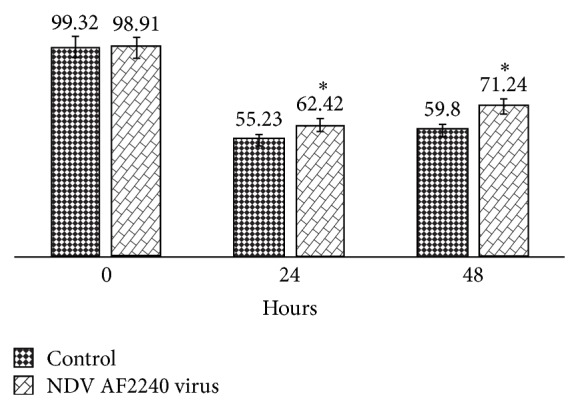
Percentage open wound area performed with TScratch software. Data are shown as % of open wound area in comparison with control cells. Cells were treated with IC_50 _(25.99 HAU/mL) value of NDV AF2240 at 0 hour (99.32%), and the results were expressed as mean ± SD from triplicate (*n* = 3) independent experiments. A significant different (^*^ *P* < 0.05) was observed when the control cells were compared with the treated cells.

Migration of cancer cells across the blood vessel-lining endothelial cells plays a significant role in cancer proliferation and metastasis. Effect of NDV AF2240 virus strain was studied on MDA-MB-231 breast cancer cells migration by using scratch wound assay. In this experiment, the virus caused a decrease in the number of cells migrating into the wound area of the MDA-MB-231 cells. The virus induced maximum inhibitory effect at 48 hours compared with control cells (0 hours), where wounds were getting healed. NDV AF2240 strain inhibited MDA-MB-231 cancer cells migration at 48 hours by approximately 29% compared with control cells (Figures 4 and 5). These results showed that, besides its antiproliferative effect, NDV AF2240 inhibited the migration of MDA-MB-231 breast cancer cell line* in vitro*. Note that the morphology of the treated cells was changing gradually with the time of exposure to the virus. The virus caused the lysis of the cells at both 24 hours and 48 hours (Figure 4), thereby making the neighboring cells ruptured and separate themselves.

### 3.4. Phase Contrast Microscopic Morphology of MD-MB-231 Cell Line

**Figure 6:**
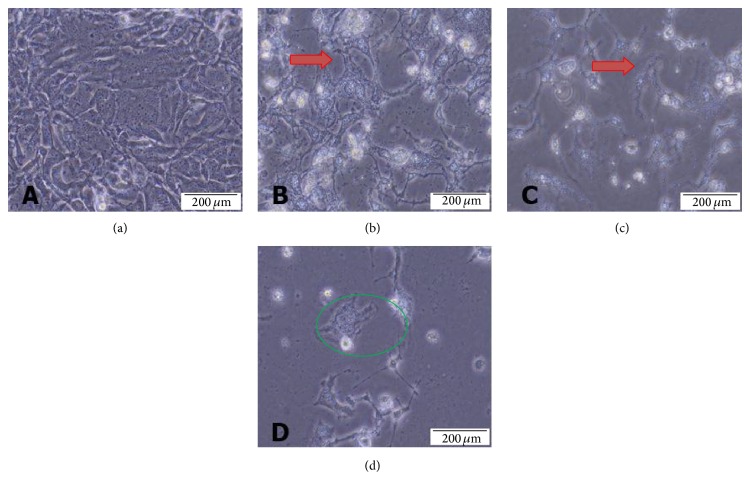
Phase contrast micrographs of MDA-MB-231 breast cancer cell lines treated with NDV AF2240 IC_50_ concentration value. (a) Untreated MDA-MB-231 cell line after (b) 24 hours (c) 48 hours, and (d) 72 hours of treatment. The virus caused the cells to lose contact with adjacent cells (red arrow) and cell membrane disruption (green circle). Mag 20x.

The effects of NDV AF2240 strain on cellular morphology of human mammary carcinoma cell line, MD-MB-231, were observed under phase contrast microscope. The observed morphological changes were recorded for both the cells treated with IC_50_ concentration value of the virus and the untreated cells at a time courses of 24, 48, and 72 hours, respectively. The treated cells showed a gradual change of cellular morphology from healthy round shining cells (Figure 6(a)) to irregular shape, sparse cell density population, in a time-dependent manner (Figures 6(b), 6(c), and 6(d)). In comparison with untreated (cancer control) cells, it was noted the appearance of cellular shrinkage and increased ruptured cells with time, indicating cellular disintegration, membrane blebbing and buds were also detected within 24 hours after treatment (Figure 6(b)). Likewise, reduction of viable cells and increase in cell debris in the background of treated cells at 48 hours and 72 hours after treatment was also detected (Figures 6(c) and 6(d)). In addition, the virus caused the confluent cells in culture to lose contact with adjacent cells, just like in cytopathic effects (CPE). All these morphological changes are signs of cellular apoptosis, a sign of cell death.

### 3.5. Flow Cytometric Analysis of DNA Fragmentation

**Figure 7:**
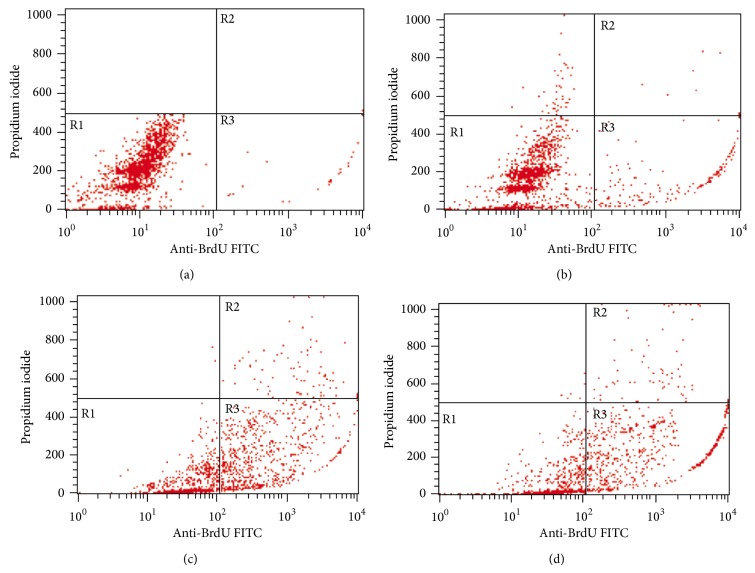
Contour diagram of BrdUTP/PI flow cytometry for MDA-MB-231 breast cancer cell lines treated with NDV AF2240 IC_50_ concentration value. (a) Untreated cells after (b) 24 hours (c) 48 hours, and (d) 72 hours of treatment. The lower left quadrants of each panel (R1) show the viable cells, which exclude PI and are negative for BrdUTP binding. The upper right quadrants (R2) contain the nonviable, necrotic cells, positive for BrdUTP binding and for PI uptake. The lower right quadrants (R3) represent the fragmented DNA (apoptotic cells), BrdUTP positive, and PI negative.

Figure 7 shows the results for flow cytometric analysis of BrdUTP/PI stained MDA-MB-231 cells posttreated with IC_50_ concentration value of NDV AF2240 strain at 24 (Figure 7(b)), 48 (Figure 7(c)), and 72 hours periods (Figure 7(d)), respectively. The lower left quadrant (R1) of the cytograms is indicating the total number of viable cells, which excluded PI and were negative for BrdUTP binding, while the upper right quadrant (R2) represents the nonviable (necrotic cells) which is positive for BrdUTP binding and showing PI uptake. The lower right quadrant (R3) represents the number of cells that had DNA fragmentation (apoptotic cells) and BrdUTP positive but negative for PI stain, indicating BrdUTP binding. The BrdUTP + PI apoptotic cell population for MDA-MB-231 cell line increased significantly (^*^ *P* < 0.05) from 1.71 ± 0.02% in untreated cells to 11.66 ± 0.91% in cells treated for 24 hours and to 36.98 ± 2.71% and 44.52 ± 10.13% in cells treated for 48 and 72 hours (Table 1), respectively.

### 3.6. Fluorescent Microscopic Morphology of MD-MB-231 Cell Line

**Figure 8:**
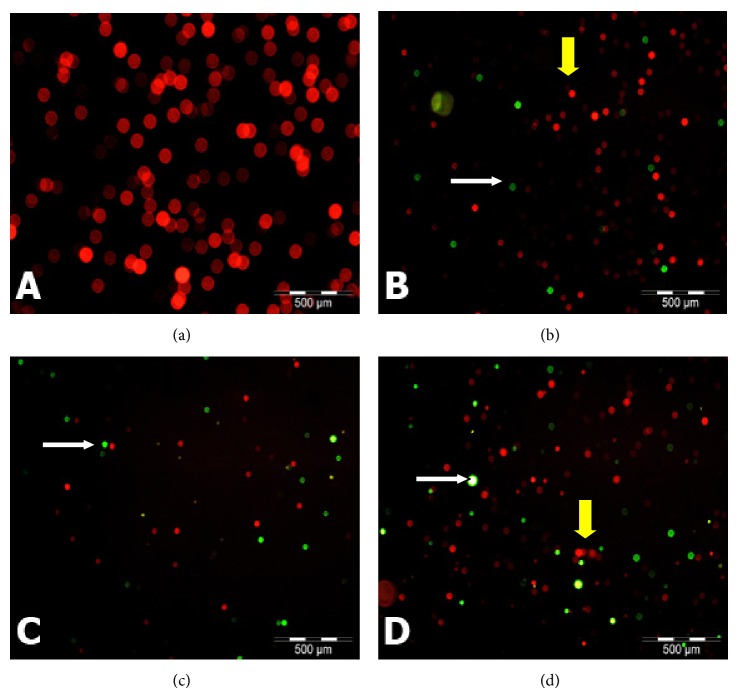
Fluorescent micrographs of MDA-MB-231 breast cancer cells treated with NDV AF2240 stained by TUNEL assay technique (Apo-BrdU), double stained with fluorescein Br-dUTP and PI/RNase A solution: (a) untreated (cancer control) cells after (b) 24 hours (c) 48 hours, and (d) 72 hours of treatment periods. Viable cells show orange-red nuclei in color (yellow arrow), whereas apoptotic cells show yellow to greenish nuclei (white arrow). Mag 20x.

**Figure 9:**
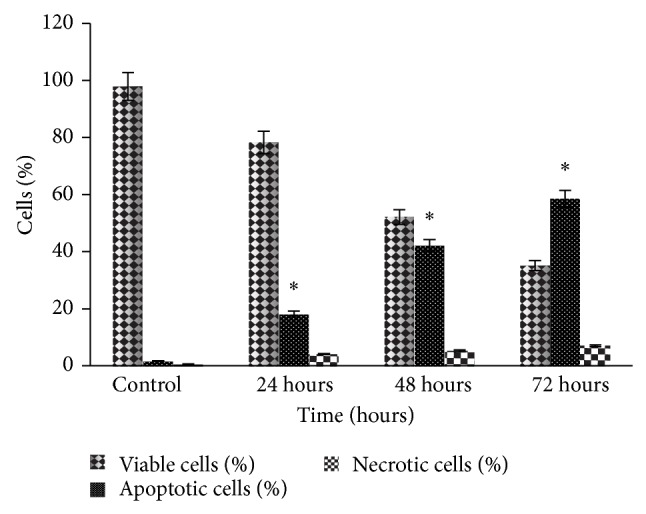
Fluorescent microscopy examinations. Percentages of viable, apoptotic, and necrotic cells in MDA-MB-231 cell population treated with NDV AF2240 after 24, 48, and 72 hours. Data shown are mean ± SD from triplicate (*n* = 3) independent experiments. Cells death via apoptosis increased significantly (^*^ *P* < 0.05) in time-dependent manner. However, no significant (*P* > 0.05) difference was observed in the necrotic cells.

Fluorescent micrographs of untreated and treated (with NDV AF2240) MDA-MB-231 breast cancer cell line showed prominent apoptotic features (Figure 8). Cells were counter stained with PI and the Br-dUTP/TdT enzyme. Apoptotic cells showed green fluorescence (color), whereas orange to green fluorescence indicated that the cells were undergoing apoptosis while the red fluorescence was an indication of viable cells (Figure 8). Based on these figures, there were no observable features of apoptotic cells seen in the untreated cancer control cells (Figure 8(a)). From the first 24 hours treatment time until the third day (72 hours) of treatment, there was significant (^*^ *P* < 0.05) increased in the number of apoptotic cells (Figure 9), as the cells are becoming more greener fluorescent (Figures 8(b) to 8(d)), more smaller in size and displayed space in between the neighbouring cells. This is indicating that the treated cells lost contacts between each other more than the untreated (cancer control) cells, while Figures 8(b) and 8(d) show that few cells are undergoing apoptosis (orange-green color). Untreated (cancer control) cells are 99% viable as shown by red fluorescence color and they also indicate the connection between each other (Figure 8(a)). Therefore, treated group showed more cells with green fluorescence in color compared with the untreated group.

### 3.7. Flow Cytometric Analysis of Cell Cycle and DNA Content

**Figure 10:**
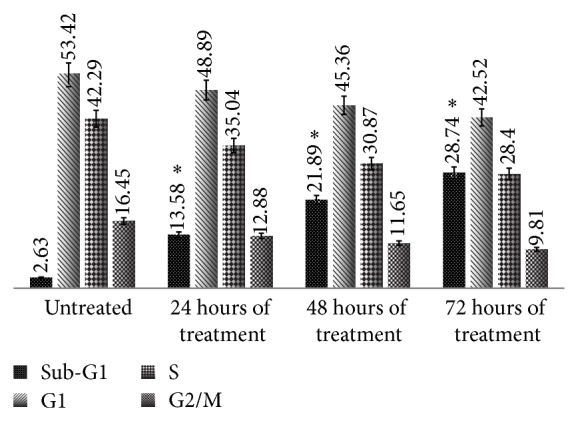
Flow cytometric cell cycle analysis of MDA-MB-231 cell after staining with BrdUTP/PI. Cells treated with NDV AF2240 strain at IC_50_. Data shown in mean ± SD from triplicate (*n* = 3) independent experiments. One-way ANOVA analysis was run with Tukey's HSD Post Hoc multiple comparison tests. Significant at (^*^ *P* < 0.05) compared with the control.

Triplicate independent experiments (*n* = 3) were performed to evaluate the effect of NDV AF2240 on DNA content by cell cycle distribution using human mammary carcinoma cells, MDA-MB-231 cell line. Cells were treated with the IC_50_ concentration value of the virus at different times as in 24 hours, 48 hours, and 72 hours and then were subjected to Apo-BrdU* in situ* DNA fragmentation assay kit's protocols. FACS analysis was used to study the cell cycle distribution (G0, G1, S, and G2/M) of the treated MDA-MB-231 breast cancer cell lines analyzed by Cell Quest Software. Results of this experiment showed that there was significant increase in hypodiploid sub-G1 region (apoptotic peak) of the cell cycle (Figure 10), in time-dependent (*P* < 0.05) of MDA-MB-231 cell treated with NDV AF2240 strain compared to untreated (cancer control) cells, signifying apoptotic induction by the virus. The percentage of the sub-G1 was 2.63% in the untreated (cancer control) cells, which increases to 13.2% after 24 hours treatment with the virus. A significant increase in apoptosis (Sub-G1) induction with time (*P* < 0.05) after 48 and 72 hours was observed (Figure 10) from 21.78% to 28.45%, respectively. However, the NDV AF2240 did not affect or induce cell cycle arrest in any specific phase of the cycle. The virus induced cell cycle unrest agreed with the previous results, suggesting that MDA-MB-231 cells undergo apoptosis more extensively with increase in time.

## 4. Discussion and Conclusions

Purified Newcastle disease virus (NDV) AF2240 strains were used to determine its oncolytic activity by observing its effects on cell viability. In the presence study, MTT assay was carried out to evaluate and study the effects of NDV AF2240 on cells viability for human mammary carcinoma cell line, MDA-MB-231, human umbilical endothelial cell (HUVECs) and human epithelial breast cell line, Hs578Bst. The assay served as a screening test to detect the inhibitory concentration (IC_50_) value of the virus that will reduce or cause 50% death of the cancer cells. The IC_50_ value obtained was 2^4.7^ (25.99) HA units/mL, demonstrating that the virus induced an efficient killing of 50% of the MDA-MB-231, cancerous cells, whereas no significant effects were observed on HUVECs and Hs578Bst (the noncancerous cells). This supports the fact that the virus does not have effect on normal cells unlike cancer cells where it showed its oncolytic effects, and this is due to the defects on the antiviral pathway on the cancer cells [28, 29]. Selectivity of the NDV to kill cancer cells has also been studied on different types of human cell lines such as osteosarcoma, bladder carcinoma, fibrosarcoma, neuroblastoma, cervical carcinoma, and Wilm's tumor using 73-T strain of the virus by Reichard et al. [14] but spared the normal human fibroblast cells. Thus, the present study demonstrated evidence that NDV AF2240 strain of the virus possesses an* in vitro* anticancer activity on MDA-MB-231 breast cancer cells with no toxic effects to normal cells such as endothelial cells, HUVECs, and epithelial breast cell line, Hs578Bst.

We further evaluated the effect of NDV AF2240 strain on cellular proliferation and the migration of MDA-MB-231 breast cancer cell line. The results showed that NDV AF2240 decreased the proliferation of MDA-MB-231 breast cancer cells in time-dependent manner. The virus inhibited cells proliferation at its maximum concentration value (IC_75_) than at its lower concentration (IC_50_). On the other hand, the untreated (cancer control) cells demonstrated an increase in proliferation rates. Therefore, the virus was able to inhibit cell proliferation. This study complied with the previous studies, which showed that NDV AF2240 strain inhibited the proliferation of other types of cancer cell lines such as brain tumor, DBTRG.05MG and U-87MG cells, and leukemia WEHI-3B cell line [19, 30], respectively. Scratch wound assay was carried out to determine its effects on cell migration* in vitro*. The result showed that virus caused a decrease in the number of cells migrating into the wound area of the MDA-MB-231 cells and induced inhibitory effect at 48 hours compared with control cells (0 hours). This indicated that, apart from its antiproliferative effect, NDV AF2240 strain inhibited the migration of MDA-MB-231 breast cancer cell line* in vitro*. Thus, this is the first evidence to indicate the effect of Newcastle disease virus on cellular migration of breast cancer cells through the use of an* in vitro* model of angiogenesis, that is proliferation and migration.

Morphological changes seen on the treated MDA-MB-231 breast cancer cells are some of the distinct features to confirm apoptosis. Fluorescence microscopic analysis of the untreated MDA-MB-231 cells showed spherical and oval to round body shape with manifestation of smooth surface, while the cells treated with NDV AF2240 strain exhibit specific morphological modification such as cells becoming smaller in size than the untreated cells, indicating cellular shrinkage, and cell membrane and chromatin condensation, rounding up of nuclei and dispersion of cells sparingly after 72 hours of treatment, demonstrating apoptosis features. This study concurred with the previous studies [31, 32], where a marked decrease in cellular growth rate of WEHI-3B leukaemic and U-87MG brain cancer cells was observed after treatment with NDV AF2240. It is inspiring to know that many NDV such as MTH-68/H, D90, and HUJ strains have been studied on glioblastoma, A549 lung cancer, and prostate cancer cells, respectively [33–35]. Two Malaysian strains, AF2240 and V4-UPM, have all been shown to be potential treatment of brain, breast, and leukaemic cancers cell lines [16, 30, 36]. Similar morphological features were also reported by the previous studies after treating cancer cells with the same virus strain AF2240 [19, 30].

The terminal transferase-mediated dUTP nick end labeling (TUNEL) assay is the most widely used technique to study apoptosis quantitatively [37]. Although the morphological changes in apoptosis are best seen by electron microscopy which remain one of the gold standard for identifying apoptotic cell, the technique may fail when standard quantitative apoptosis count is required [38, 39]. The greatest sensitivity of the TUNEL assay technique to label and detect the DNA breaks has led to its wide acceptance and use in most of the major laboratories. This assay was used in this study to confirm apoptosis by flow cytometry that analyzed the cells population and quantified the DNA strand breaks (DSBs). The results showed that the Brd-UTP + PI apoptotic cell population for MDA-MB-231 cell line increased significantly (*P* < 0.05) from 1.71 ± 0.02% in untreated cells to 11.66 ± 0.91% in cells treated for 24 hours. The untreated (cancer control) cells were having intact DNA or the fragmented DNA in them was too low to be detected and bounded by the BrdUTP fluorochromes dye as it was shown in the contour cytograms above. This is the first study to quantify the fragmented DNA due to NDV AF2240 strain by flow cytometry, instead of agarose gel electrophoresis qualitatively.

Meanwhile, there are many studies that used the TUNEL assay techniques in different perspective sto quantify apoptosis by fluorescence microscopy [15, 40]. Evidence has shown that Br-dUTP is more readily incorporated into the genome of apoptotic cells than are the deoxynucleotide triphosphates complexes to larger ligands such as fluorescein, biotin, and/or digoxigenin [41, 42]. Thus, the assay utilizing Br-dUTP is more sensitive in terms of detecting DNA strand break of apoptotic cells. In addition to quantification of the fragmented DNA in the apoptotic cells, quantitative analysis of cell cycle is essential in the study of molecular mechanism of cell death [43]. Based on this principle, a flow cytometric analysis of cell cycle was studied to observe the effects of the NDV AF2240 strain on DNA content and the phases of cell cycle. Analysis of the treated MDA-MB-231 breast cancer cell lines after being double stained with PI versus Br-dUTP revealed an increase in the sub-G1 region of the cell cycle with time as compared with the untreated (cancer control) cells (*P* < 0.05), indicating apoptosis induction by NDV AF2240 with no detection of cell cycle arrest by the virus. Study of cell cycle pattern has documented that NDV AF2240 strain caused an increase in the apoptotic peak (sub-G1) with no arrest in any of the cell cycle phases [16, 30]. These results agreed with the previous studies that suggest that NDV AF2240 induced apoptosis to cancer cells more extensively with an increase in time.

Based on this study, we can conclude that the NDV AF2240 strain showed a remarkable potency as an anticancer agent on MDA-MB-231 human breast cancer cells. Its oncolytic properties and ability to induce apoptosis in breast cancer cells indicated that the virus could be the best choice for future treatment of breast cancer patients. Although, the mechanism by which it induces its anticancer activity is not completely understood, further studies need to be carried out to complete the preclinical requirements. Our results show that NDV AF2240 is a promising anticancer agent in virotherapy application for the management of breast cancer.

## Figures and Tables

**Table 1:** Mean ± SD of percentages of DNA strand break of apoptotic cells (DNA fragmentation) of MDA-MB-231 negative control treated with NDV AF2240 at different time intervals.

Cell	Time of treatment	% of DNA fragmentation (apoptotic cells)
MDA-MB-231	Control cells	1.71 ± 0.02
	24 hours of treatment	11.66 ± 0.92^*^
	48 hours of treatment	36.98 ± 2.71^*^
	72 hours of treatment	44.52 ± 10.13^*^

^*^Significant (*P* < 0.05) compared to untreated (cancer control) cells.
